# A descriptive study on the treatment of pediatric CRPS in the Nordic countries and Germany

**DOI:** 10.1002/pne2.12064

**Published:** 2021-11-14

**Authors:** Johanna Broman, Cathrin Weigel, Ludwig Hellmundt, Anna Persson

**Affiliations:** ^1^ Department of Clinical Sciences Lund/Helsingborg, Anesthesiology and Intensive Care Medicine Lund University Lund Sweden; ^2^ Department of Anesthesiology and Intensive Care Medicine Helsingborg Hospital Helsingborg Sweden; ^3^ Department of Anesthesia and Intensive Care Children's Hospital "Auf der Bult" Hannover Hannover Germany; ^4^ Department of Anesthesia and intensive Care Pain Unit Södertälje Hospital Södertälje Sweden; ^5^ Department of Clinical Sciences Malmö, Anesthesiology and Intensive Care Medicine Lund University Lund Sweden; ^6^ Department of Anesthesiology and Intensive Care Medicine Hallands Hospital Halmstad Halmstad Sweden

**Keywords:** CRPS, pain centers, pCRPS, persistent pediatric pain

## Abstract

Pediatric complex regional pain syndrome (pCRPS) is a rare, painful state that often occurs as a complication following physical trauma. Diagnosis and treatment require specialist expertise in a multidisciplinary setting. Treatment is focused on pain reduction and improvement in function, which differs from the treatment of adult CRPS. We performed a cross‐sectional survey with the aim of identifying pain centers in the Nordic countries and Germany that specialized in treating children with pain, especially pCRPS, and sought to describe their treatment strategies. Centers and health‐care professionals working with children experiencing chronic pain were identified using internet search engines, phones, or e‐mail. A standardized set of questions and an electronic questionnaire were answered by the participants. A total of 28 participants were identified in 24 centers, which were involved with patients having pCRPS (Germany: 7, Norway: 7, Sweden: 5, Finland: 5, Denmark: 3, and Island: 1). One center in Germany treated more than 20 patients per year. Half of the identified centers (n = 12) treated between 1 and 5 children with pCRPS per year. Guidelines for treating pCRPS were reportedly followed by 9/28 responders (32%), and physiotherapy was reported to be part of the treatment routine in most centers (74%). Interventional anesthesia was rarely used. Psychological therapy: 57% answered that it was always offered, 30% replied that it was proffered in most cases, and 13% responded that it was recommended in only a few patients. Pharmacological treatments were not commonly used. Treatment resources for pCRPS are scarce in the Nordic countries and Germany. Most centers treated very few children with pCRPS and did not have established guidelines. A multidisciplinary approach was used by many centers, most often combining physiotherapy and psychotherapy, and less commonly pharmacological treatment. The difficulties in diagnosing pCRPS and finding official referral units are unfortunate, considering the potentially favorable outcome with adequate treatment.

## 1 INTRODUCTION

Complex regional pain syndrome (CRPS) is a rare cause of pain that is typically located in the hand or foot, combined with symptoms of the sensory, motor, and autonomic nervous systems.^1^, ^2^ It is characterized by the continuous presence of pain that is out of proportion to the medical history or findings on physical examination. Presentation in children and adolescents differs from adult CRPS,^1^, ^2^ with children usually presenting with burning pain in a single limb (most commonly in a lower extremity), along with changes in autonomic symptoms that includes temperature, edema, and hyperhidrosis.^3^ The mean age of pediatric CRPS (pCRPS) is approximately 12 years, and female adolescents are predominantly affected. pCRPS is unusual in children younger than seven years of age.^3^ The specific causes are largely unknown, while diagnostics and treatment options are currently debated. However, a multidisciplinary approach is generally recommended.^4^ The trigger can be a relatively minor trauma,^2^ but in some cases, no previous injury is identified.^1^ A high rate of comorbid psychological disorders and stress are believed to play an important role in inducing or perpetuating pCRPS.^1^ Clinical evaluation using adult criteria remains the gold standard for diagnosis of CRPS.^1^ The definition of CRPS follows the Budapest criteria developed in 2004 and adopted by the International Association for the Study of Pain (IASP) (Table 1).

**TABLE 1:** Diagnostic criteria as defined by the International Association for the Study of Pain (IASP) 2004

Diagnostic criteria for complex regional pain syndrome type 1 (Budapest, 2004)
1. Continuing pain, disproportionate to any inciting event
2. At least one symptom in three of the following four categories:
Sensory: history of hyperalgesia and/or allodynia
Vasomotor: history of temperature asymmetry and/or skin color change and/or skin color asymmetry
Sudomotor/edema: history of edema and/or sweating changes and/or sweating asymmetry
Motor/trophic: history of decreased range of motion and/or motor dysfunction (weakness, tremor, and dystonia), and/or trophic changes (hair, nails, and skin).
3. During the evaluation, at least one sign in two or more of the following four categories:
Sensory: evidence of hyperalgesia (to pinprick) and/or allodynia (to light touch and/or deep somatic pressure and/or to joint movement)
Vasomotor: evidence of temperature asymmetry and/or skin color changes and/or asymmetry
Sudomotor/edema: evidence of edema and/or sweating changes and/or sweating asymmetry
Motor/trophic: evidence of decreased range of motion and/or motor dysfunction (weakness, tremor, and dystonia) and/or trophic changes (hair, nails, and skin).
4. There is no other diagnosis that better explains the signs and symptoms

A multidisciplinary approach to treatment, focused on reducing pain and improving physical function, is advised.^3^ Both the child and the surrounding environment (family, academic, and social) should be taken into consideration.^1^ Physical and occupational therapy is recognized as successful treatment strategies.^5^ However, there is no consensus regarding the duration, intensity, or specific content of the treatment.^1^ Medication(s) could be beneficial although pharmacological treatment of pCRPS is less common in children due to the favorable response to physical and occupational therapy.^3^ To date, there are very few randomized clinical trials about pharmacological interventions for treating pCRPS, with the type of analgesics used often varying between clinicians and institutions. Pharmacological treatment is predominantly aimed at achieving analgesia.^6^ However, medications can also be used to treat the associated symptoms of pCRPS, such as anxiety and sleep issues.^3^

With intensive treatment, the prognosis of pCRPS has improved substantially and is now considered to be favorable.^1^ However, little is known about the diagnostic and treatment resources for pCRPS in northern Europe. The Scandinavian Society of Anaesthesiology and Intensive Care Medicine initiated this study in the fellowship program for pediatric anesthesia and intensive care, with centers from the Nordic countries and Germany chosen as the first attempt to identify diagnostic and treatment resources for pCRPS in northern Europe. The scarcity of organizations dealing with this important issue could affect outcomes, considering the need for intensive, multidisciplinary treatment. Hence, the aim of this study was to identify pain centers in the Nordic countries and Germany that specialized in treating children with pain, especially pCRPS, and to describe their treatment strategy.

## 2 METHODS

### 2.1 Study design

We conducted a descriptive study based on a cross‐sectional survey carried out in 2019. The analysis was performed in three sections. First, we aimed to identify dedicated medical personnel working with children diagnosed as having pCRPS. Second, we asked participants a few short questions regarding the treatment approach for pCRPS (as specified below). Third, the participants were asked to complete a questionnaire.

### 2.2 Study setting/Primary survey

We contacted pain centers connected to children's hospitals in the Nordic countries and Germany initially, aiming to find a dedicated person from the center, who treated children with CRPS. We included pediatric pain centers that treated patient referrals with a diagnosis of pediatric pain. We sought to contact the person responsible for the treatment of pCRPS via e‐mail or telephone and asked the following questions:

1. What is your profession?
2. Do you treat children with CRPS? Yes/No
3. How many patients with CRPS do you treat per year?
4. Do you know of anyone else in your region who treats these children?
5. Could you participate in a survey regarding treatment of pediatric CRPS?

We asked if they knew of other centers that treated chronic pain in children, specifically CRPS, and then contacted them. We excluded participants who did not treat children with CRPS.

### 2.3 Questionnaire/ secondary survey

**FIGURE 1:**
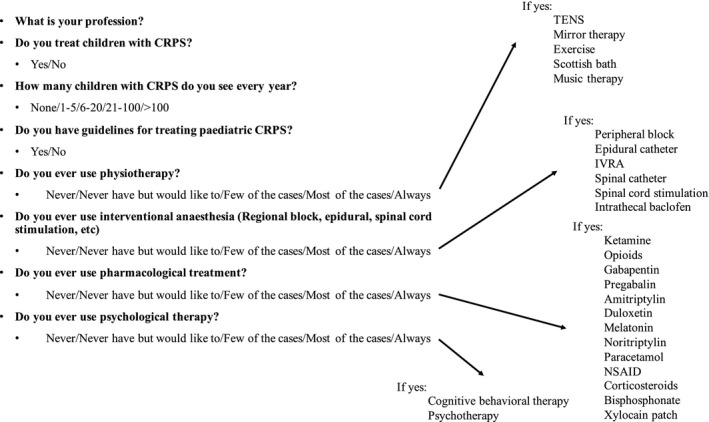
Questions surveyed in the questionnaire

A questionnaire regarding the treatment of CRPS was developed by the authors with a goal to incorporate all known and plausible pharmacological and interventional treatments for pCRPS, including physiotherapy and psychotherapy. Questions regarding general psychological treatments were probed (Figure 1). The questionnaire was constructed using the survey tool esMaker NX3 (Entergate, Halmstad, Sweden).

### 2.4 Data collection

A link to the electronic survey was sent out to all identified professionals involved in treating pCRPS via e‐mail. A reminder was sent out few weeks later and at two months after the first notification in an attempt to increase the response rate. Participants who did not respond to the electronic questionnaire were contacted again via e‐mail to obtain their responses regarding the additional questions (whether physiotherapy/ psychological/ pharmacological/ interventional treatments were used, and how often these were employed: never/ have never but would like to/ in few cases/ in most cases/ always).

### 2.5 Data analysis/ statistics

Basic descriptive statistics were used to analyze the data. The number of involved centers and type of data did not permit the use of more advanced statistical analysis. Data were analyzed using IBM SPSS software (version 26.0; IBM Inc).

### 2.6 Ethical considerations

Since the study was performed as part of a student research project and was descriptive in nature, the need for ethical approval was waived. The participation of professionals working with children experiencing pain was voluntary, and the survey was conducted anonymously. None of the patients were included in the study. No sensitive personal data were included in this study.

## 3 RESULTS

### 3.1 Primary survey—number of patients and use of guidelines

**FIGURE 2:**
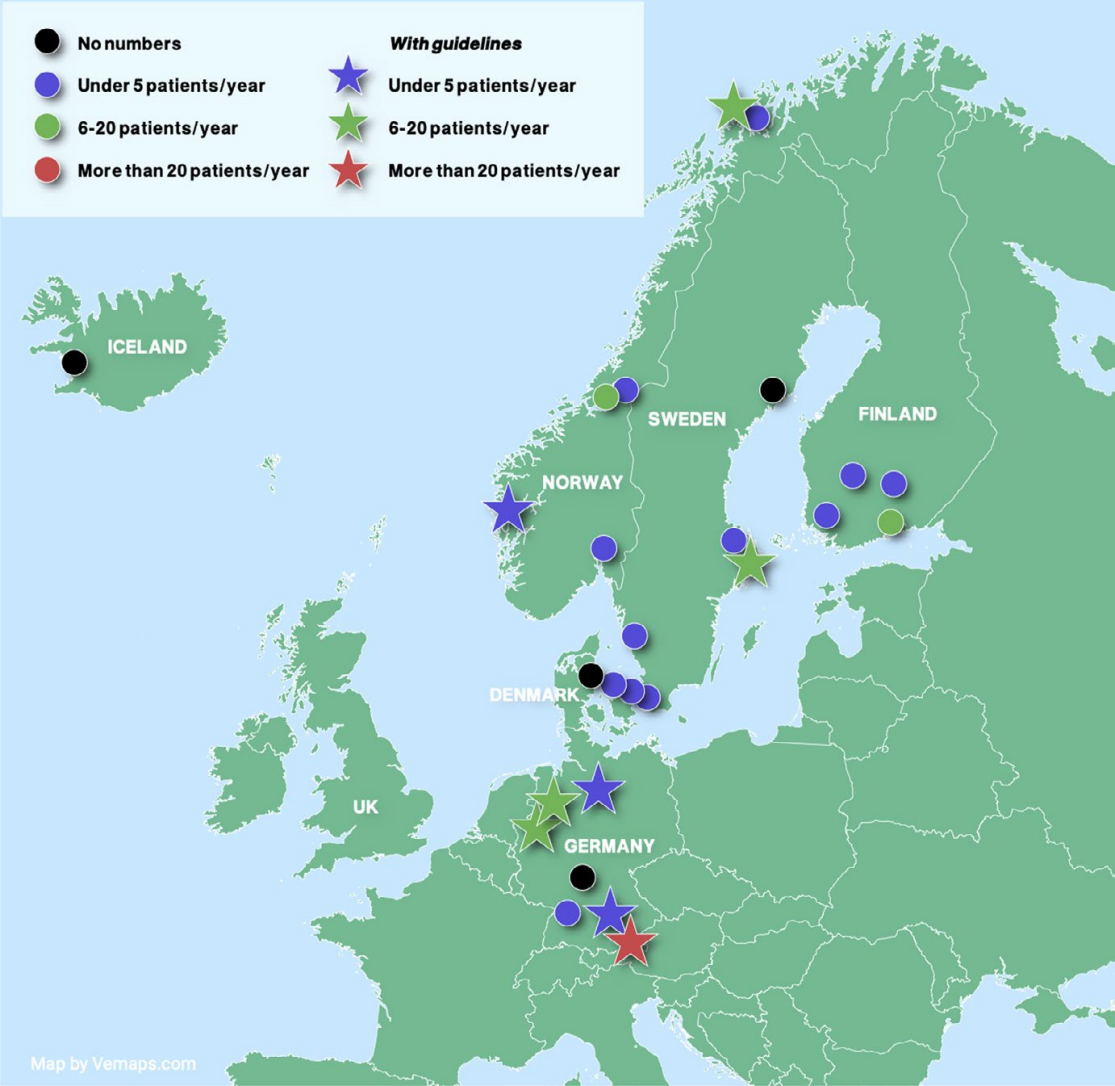
Map demonstrating the inclusion of participants working with children diagnosed as having pCRPS

A total of 28 participants working with children diagnosed as having pCRPS were identified at 24 centers (Germany: 7, Norway: 7, Sweden: 5, Finland: 5, Denmark: 3, and Island: 1). Many centers seemed to have a dedicated person working with these rare patients. A few centers had more than one person from differing professions, working at different clinics, and hence, we included both responders. Thus, there were four more participants than the number of centers. Twenty‐five participants answered the primary survey regarding the number of pCRPS patients and treatment guidelines with a response rate of 89%. Fourteen responders were reported to be anesthesiologists: five were pediatricians, one was a pain physician, three were physiotherapists, one was a rehabilitation doctor, and another was a general practitioner. There were no patients with pCRPS under the care of three responders, even though they treated children with other types of persistent pain. Hence, they did not answer the primary survey. Fifteen responders treated between 1 and 5 children with pCRPS per year. Six responders reported treating 6‐20 children with pCRPS annually. One responder in Germany treated more than 20 children per year. Nine responders reported having written treatment guidelines (Figure 2, Table 2).

**TABLE 2:** Number of centers treating pCRPS and the estimate of the number of pCRPS patients per year in each center and the use of guidelines at these centers

Country	No. of identified centers	Patients with pCRPS/center/year	Guidelines
Sweden	5	1‐20	1/5 (20%)
Norway	7	0‐20	2/7 (29%)
Island	1	0	0
Finland	5	1‐30	0
Denmark	3	1‐5	1/3 (33%)
Germany	7	1‐100	5/7 (71%)
	Total number: 28		

### 3.2 Secondary survey—treatment approach

Nineteen participants completed the secondary survey (Figure 1) and the electronic questionnaire. Two participants denied treating patients with pCRPS and thus did not answer further questions. Six of them did not answer the electronic questionnaire but were contacted again via e‐mail or phone, who answered questions regarding the four treatment groups in general, but did not answer specific questions regarding the different therapy options (n = 25). Regarding specific therapies, few questions were not answered by some responders.

### 3.3 The types of physiotherapy used

Among the participants, 74% used physiotherapy in all patients and the remaining used it in most cases (13%) or few cases (13%). Regarding the different types of physiotherapy recommended for use (exercise, TENS—transcutaneous electrical nerve stimulation, mirror therapy, Scottish bath—alternating hot and cold streams of water during a shower, and music therapy), the answers about the preferred choice differed widely among the respondents except for exercise, which was used by all (Table 3).

**TABLE 3:** Responses regarding different treatment options used while treating pCRPS

	Never	Have never but would like to	Few of the cases	Most of the cases	Always	N
**Physiotherapy**			**3**	**3**	**17**	**23**
TENS	6	1	6	3		16
Mirror therapy	4	3	5	2	2	16
Exercise				5	11	16
Scottish bath	11	1	1	1	2	16
Music therapy	5	4	3	4		16
**Pharmacological treatment**	**5**		**13**	**3**	**2**	**23**
Ketamine	9		6			15
Opioids	12		3			15
Gabapentin	3		9	3		15
Pregabalin	5		8	1		14
Amitriptyline	3		10	2		15
Duloxetine	12		3			15
Melatonin	5		8	2		15
Nortriptyline	8		6	1		15
Paracetamol	5		6	4		15
NSAID	5		7	3		15
Corticosteroids	13	1	1			15
Bisphosphonates	15					15
Xylocaine patch	9	1	4	1		15
**Interventional therapy**	**15**		**8**			**23**
Peripheral blocks	13		2			15
Epidural blocks	11		4			15
Spinal blocks	15					15
Spinal cord stimulation	14		1			15
Intrathecal baclofen	15		1			16
IVRA	15		1			16
**Psychological therapy**			**3**	**7**	**13**	**23**
CBT			2	7	7	16
Psychotherapy	1		7	4	3	15

Note

Highlighted text involves general questions asked. Questions regarding specific therapies were not answered by all.

### 3.4 Pharmacological treatment preferred

Pharmacological treatment options were never considered by 22% of the respondents. However, 56% used them in few cases, 13% in most cases, and 9% used them in all cases. The drugs reportedly being used were as follows: gabapentin and amitriptyline in 80%; pregabalin, paracetamol, NSAID, and melatonin in 67%; and ketamine in 40%. None of the respondents reported using only one specific drug. Several respondents denied the use of any of the above‐mentioned drugs (Table 3).

### 3.5 Psychological therapy offered

More than half (57%) of the respondents offered psychological therapy for all patients, 30% offered therapy to most patients, and 13% employed it in a few cases. All respondents reported the use of both cognitive behavioral therapy (CBT) and psychotherapy at varying frequencies (Table 3).

### 3.6 Interventional therapy implemented

The use of interventional therapy was only reported by a few participants (65% had never used it, while 35% had employed it in a few cases). Epidural injections and peripheral blocks were used in a few patients. Spinal cord stimulation, intrathecal baclofen, and intravenous regional anesthesia (IVRA) were administered only by one respondent (Table 3).

## 4 DISCUSSION

We experienced difficulties finding referral centers with resources to care for patients with pCRPS. In addition, treatment resources and approaches varied, and there was no central authority regulating guidelines for this patient group. However, we found individuals and dedicated pain centers with special interest in pCRPS using contacts. After communicating with these contacts, we concluded that there was no consensus regarding the treatment of pCRPS in the Nordic countries and Germany. Only 9 out of 28 participants, who responded to our survey, utilized guidelines when treating children with CRPS.

Persistent pain in children is uncommon but may have a negative impact on a child's quality of life and development.^6^ The incidence of pCRPS in children in the Nordic countries is unknown. In one study, the incidence was 1.16 per 100 000 children between the ages of 5 and 15 years.^7^ In this study, some countries reported very few cases of pCRPS, annually. Half of the centers treated fewer than 5 children per year with pCRPS. This is in line with other studies in the literature, where smaller number of reported pCRPS cases made achieving appropriate sample sizes in scientific studies unfeasible. Additionally, most physicians do not encounter large number of patients with pCRPS.^7^

The literature is reasonably consistent regarding the most effective management of these children. Accurate and early diagnosis and treatment in a multimodal setting are recommended.^2^, ^8^ However, very little evidence‐based data exist to guide the treatment of pCRPS, and there are no randomized controlled trials analyzing the multidisciplinary approach.^9^ Early diagnosis helps in preventing excessive medical testing and reduces the psychological burden of disease and is, therefore, regarded crucial for favorable outcomes. The use of Budapest criteria developed by the IASP (Table 1) is strongly recommended, even though the criteria is intended for adults.^2^, ^8^ A report from 2012 demonstrates that the incidence of CRPS after limb trauma depends on the diagnostic criteria used. Among 596 adult participants, only 7% were diagnosed with CRPS according to the current IASP criteria, while 49% were diagnosed with CRPS using the former IASP criteria, and 21% were diagnosed with CRPS using the criteria often employed by surgeons.^10^ The same can be expected in the pediatric population. The diagnostic criteria used for pCRPS are far from being well recognized by many physicians who care for these children. Consequently, many children with chronic pain are underdiagnosed, especially considering the difficulty of finding referral centers.

After being diagnosed with pCRPS, it is important that these children receive appropriate treatment. International guidelines suggest intensive treatment by multidisciplinary teams with weekly schedules. Physical therapy, psychological support, and pharmacological treatment should be used concurrently to supplement each other. Our study showed that physiotherapy was frequently used by all centers. This is in line with the literature, where pharmacological intervention is often described to play a secondary role in physiotherapy.^3^, ^11^ Psychological therapy was also commonly used by most centers.

There was heterogeneity in the use of physiotherapy techniques although exercise was the most common one utilized in all centers. Other suggested therapies (TENS, mirror therapy, Scottish bath, and music therapy) were occasionally used. The efficacy of these techniques has not been assessed in pediatric patients. No studies have compared management with or without rehabilitation, as this design would be considered ethically unacceptable. However, these methods seem to be the most widely used treatments for pCRPS and seem to provide good results.^2^

In terms of pharmacological treatment, there was a wide range of answers, and 20% claimed that they did not use pharmacological treatments. The drugs most commonly used in our cohort were gabapentin and amitriptyline in 80% of patients. In contrast, pregabalin, paracetamol, NSAID, or melatonin were used by two out of three respondents. When selecting drugs for our survey, we used medications that were described for use in the treatment of pCRPS in the literature.^2^, ^3^ In addition, opioids and glucocorticoids were added to this list, despite not being described in the literature as they are commonly used for treating other types of pain.^3^

The treatment of pediatric chronic pain should focus on reducing pain and improving function. In general, pharmacological treatment for CRPS is less common in children because there is good response to nonpharmacological treatment, and the concern that the risks of medication use outweigh the benefits. When used, pharmacological treatment is aimed at achieving analgesia to increase participation in physiotherapy and other activities.^1^ Medication is also commonly used to treat the associated symptoms of pCRPS, such as anxiety and sleep issues. There are no randomized clinical trials of medications for pCRPS, and medication choice often varies between clinicians and institutions. The results from our survey seem to be in line with the findings in the literature.^1^, ^3^, ^11^, ^12^, ^13^

Interventional therapies (regional block, epidural, spinal cord stimulation, etc) are not commonly used in the treatment of pCRPS, and trials on use in the pediatric population are limited. Our study reveals that, in the Nordic and German centers, it was used sparingly in very few patients. These therapies are considered as an option only after a reasonable time (four–five weeks) has passed without successful results despite intensive multimodal therapy. The type of technique that should be utilized is not based on empirical data. To the best of our knowledge, no randomized controlled trials have compared the conservative and invasive management strategies in this particular group of patients.^8^, ^14^

This study has some limitations. First, the questionnaire used in the study was author‐developed, and as the authors were anesthesiologists, the questions may have been biased. Anesthesiologists prefer pharmacological treatment more than physiotherapy and psychotherapy; hence, the questions were more detailed in this category. We welcomed other suggestions, and some responders recommended using therapies that were not being primarily offered. For example, interdisciplinary multimodal treatment, trauma psychotherapy, family therapy, social intervention (school, etc), pain education, acceptance and commitment therapy (ACT), exposure, graded motor imagery, compression textiles, lymph therapy, sensitivity therapy, exercise in pool, dog‐assisted therapy, mindfulness, art therapy, dance therapy, and clonidine (one of the drugs accepted for use).

Second, we did not ask about the specific criteria used to diagnose pCRPS. This would have been interesting, considering the findings in the above‐mentioned study, wherein the incidence varied widely depending on the criteria used.^10^ In addition, we may have missed few referral centers that were not included in the study as we experienced great difficulties in identifying them.

## 5 CONCLUSION

Most centers treating pediatric pain treated very few children with pCRPS, and guidelines were not generally used. A multidisciplinary approach, most often combining physiotherapy and psychotherapy, and less commonly pharmacological treatment or interventional therapies, was used by most physicians in accordance with the literature. However, not all pediatric pain centers seemed to have access to physiotherapists and psychologists. In the Nordic countries and Germany, treatment resources for pCRPS appeared to be scarce.

There are international recommendations regarding treatment of pCRPS. Our results implies that, despite limited means, patients are being treated according to recommendations.

## CONFLICT OF INTEREST

The authors declare no conflicts of interest.

## ETHICAL APPROVAL

Due to the arrangement of this study as a developmental project performed within the scope of an international educational program, with no experiments performed and no study subjects used, the need for ethical review by the National Ethical Review Board was waived. All participating participants provided oral consent, and data were handled anonymously.

## INFORMED CONSENT

Informed consent was obtained from all the individuals included in this study.
